# Genetic diversity of *Lepista nuda* (Agaricales, Basidiomycota) in Northeast China as indicated by SRAP and ISSR markers

**DOI:** 10.1371/journal.pone.0202761

**Published:** 2018-08-27

**Authors:** Jing Du, Hong-Bo Guo, Qi Li, Adrian Forsythe, Xu-Hui Chen, Xiao-Dan Yu

**Affiliations:** 1 College of Biological Science and Technology, Shenyang Agricultural University, Shenyang, Liaoning, China; 2 College of Life Engineering, Shenyang Institute of Technology, Fushun, Liaoning, China; 3 Department of Biology, McMaster University, Hamilton, Ontario, Canada; Mizoram University, INDIA

## Abstract

*Lepista nuda* is a popular wild edible mushroom that grows in China. In this study, we used ISSR and SRAP molecular markers to analyze the genetic diversity of 72 samples of *L*. *nuda* from eight populations in Northeast China. In total, six ISSR primers and five pairs of SRAP primers that produced clear and polymorphic banding profiles were selected for assessing *L*. *nuda* genetic diversity. The results revealed a high level of genetic variation among the 72 samples (94.4% polymorphism) but a low degree of gene flow among the populations. Among *L*. *nuda* populations, genetic distance was not correlated significantly with geographic distance. The antioxidant activity of the samples from each population was also tested and the result showed that all the selected samples had more than 60% DPPH scavenging activities. Nonetheless, the antioxidant activity diversity is not coincident with both the genetic diversity and the geographic distribution. The results indicate that ISSR and SRAP molecular markers are useful for studying the genetic diversity of *L*. *nuda*. The results also suggest that *L*. *nuda* populations in Northeast China require protection.

## Introduction

*Lepista nuda* (Bull.) Cooke is a popular edible mushroom in China [1], and natural populations are common throughout Northeast China. The species also grows naturally in Europe and North America but has not been grown commercially [2, 3]. *Lepista nuda* can be distinguished from other species by its lilac to purple-pink pileus, its white to pale-pink spore print, and its distinctive odor [3, 4]. This fungus is considered to be delicious by humans, and its fruiting bodies are also nutritious in that they contain high levels of proteins [5] and polysaccharides [6]. In addition, *L*. *nuda* extracts can inhibit the *in vitro* formation of biofilms by multi-drug-resistant bacteria [7].

In recent years in China, wild specimens of *L*. *nuda* have been extensively collected for their commercial value, and the habitat of the species has been frequently destroyed [8], suggesting that the species may be endangered in the country. Information on the genetic diversity of an endangered species can provide insight into its genetic health [9] and perhaps into its conservation, domestication, and breeding. To date, however, little is known about the population genetics of *L*. *nuda*. An analysis of ITS sequences of 66 samples of *L*. *nuda* revealed a low level of genetic diversity, but these samples were collected from only one site in China [10].

DNA fingerprinting methods have been widely used to study the genetic diversity of fungi. These methods, including the use of ISSR (inter-simple sequence repeats) [11] and SRAP (sequence-related amplified polymorphism) [12], have proven to be useful for evaluating the genetic diversity of edible mushrooms such as *Auricularia auricula-judae* (Bull.) Quél. [13], *Lentinula edodes* (Berk.) Pegler [14], *Pleurotus citrinopileatus* Singer [15], *Pleurotus eryngii* (DC.) Quél. [16], *Pleurotus pulmonarius* (Fr.) Quél. [17], and *Tricholoma matsutake* (S. Ito & S. Imai) Singer [18]. Antioxidants play an important role to maintain the cell functioning and integrity of the cells. They can help neutralize the damaging free radicals of the human body. It has been reported that certain types of mushroom possess antioxidant properties [19, 20]. Previous studies have shown that the sporophore of *L*. *nuda* has obvious antioxidant activity [21, 22]. In the current study, ISSR and SRAP markers were used to investigate the genetic diversity of eight natural populations of *L*. *nuda* in Northeast China. In addition, relationship between antioxidant capacity and genetic diversity of each population was studied.

## Materials and methods

### Ethics statement

*Lepista nuda* is neither protected nor endangered in the sampled areas, and all samples were collected by researchers following current Chinese regulations. None of the sampled locations are privately owned or protected by law.

### Sampling

**Fig 1:**
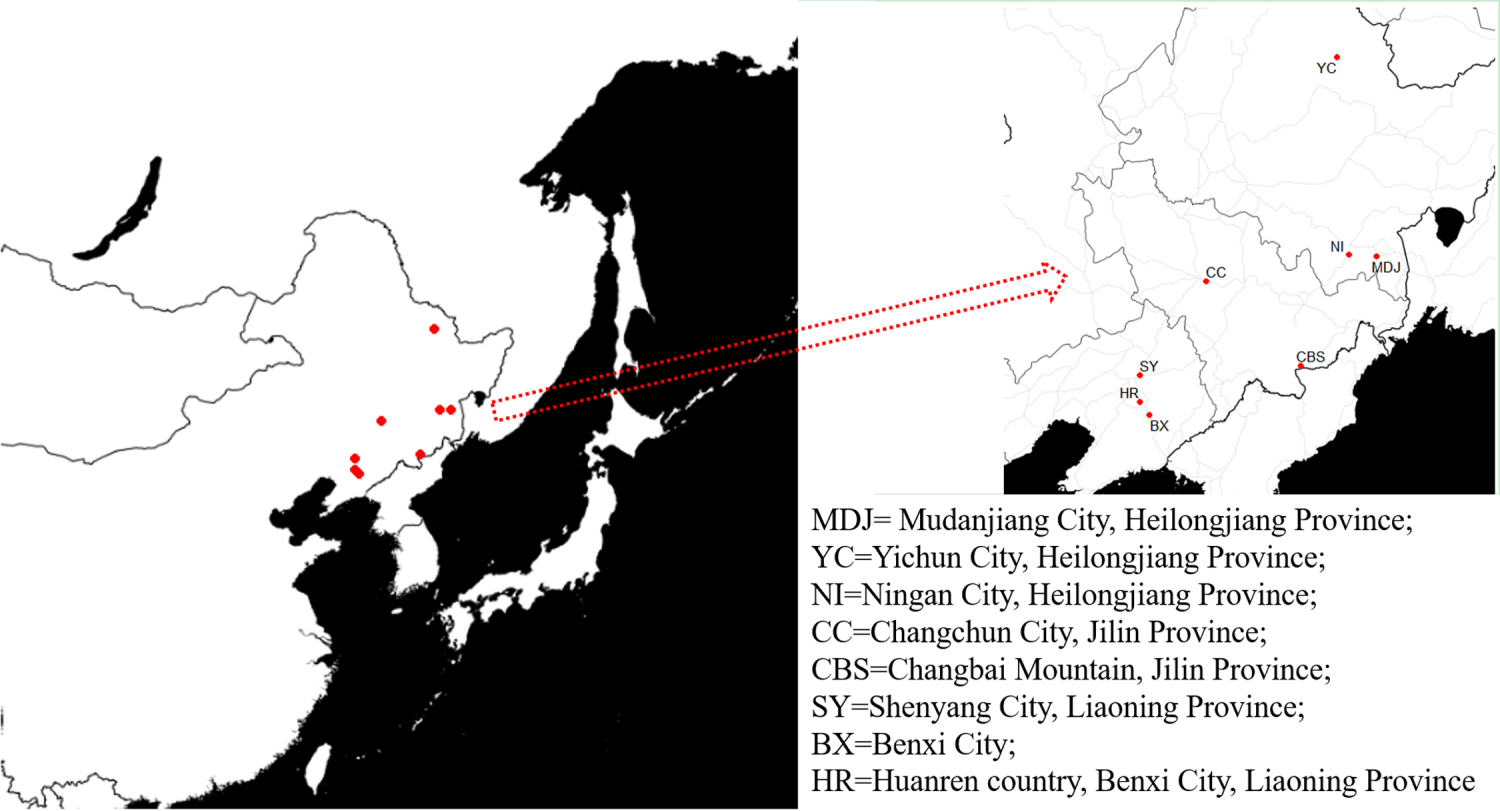
Sampling sites of *Lepista nuda* in Northeast China. Background information on the populations indicated in the right panel is provided in Table 1.

A total of 72 samples (basidiomata) were collected from eight sites in Northeast China from September 2012 to August 2015 (Fig 1). Sampling sites of *Lepista nuda* were drew by R Statistical Software [23] and packages ggplot2 and ggmap [24]. The sample size, the geographical coordinates and the types of climate and forest [25, 26] for each site are listed in Table 1. Tissue blocks were removed from the inner part of the fresh basidiomata; the blocks were dried with silica gel for DNA analyses.

**Table 1:** Sample sizes (number of basidiomata), locations, the type of climate and forest of eight *Lepista nuda* populations. A total of 72 basidiomata were collected.

Population^a^	Sample size	Latitude (north)	Longitude (south)	Climate^b^	Forest type	Voucher collection^c^
MDJ	14	44.32	130.33	DWb	Mixed forest	SYAU-FUNGI-014
YC	8	48.24	129.22	DWb	Mixed forest	SYAU-FUNGI-015
NI	4	44.35	129.56	DWb	Mixed forest	SYAU-FUNGI-016
CC	9	43.81	125.49	DWa	Mixed forest	SYAU-FUNGI-017
CBS	7	42.04	128.18	DWb	Mixed forest	SYAU-FUNGI-018
BX	9	41	123.87	DWa	Broadleaf forest	SYAU-FUNGI-019
HR	10	41.27	123.59	DWa	Broadleaf forest	SYAU-FUNGI-020
SY	11	41.84	123.59	DWa	Broadleaf forest	SYAU-FUNGI-021

a: MDJ = Mudanjiang City, Heilongjiang Province; YC = Yichun City, Heilongjiang Province; NI = Ningan City, Heilongjiang Province; CC = Changchun City, Jilin Province; CBS = Changbai Mountain, Jilin Province; SY = Shenyang City, Liaoning Province; BX = Benxi City; HR = Huanren country, Benxi City, Liaoning Province.

b: DWa = warm continental climate; DWb = Temperate continental climate, according to Köppen–Geiger climate classification system

c: Mycological Herbarium of Shenyang Agriculture University

### Identification of samples

Genomic DNA was extracted from the dried tissue blocks using a modified cetyltrimethyl ammonium bromide (CTAB) method [27]. The extracts were treated with 5 μl of RNase (10 mg/ml) in a 37°C water bath for 1 h to remove RNA. The purity and quality of the genomic DNA were determined via spectrophotometry and electrophoresis on a 1.0% agarose gel. The DNA solution was stored at -20°C. One sample was then randomly selected from each population, and the ITS regions of these samples (eight in total) were then amplified and sequenced to determine whether the collected fungi were *Lepista nuda*. Primers ITS5/ITS4 [28] were used for amplification of the ITS region including ITS1, 5.8S, and ITS2. Amplification reactions were performed in a PCR Amplifier (BIO-RAD S1000, Hercules, CA, USA) in 25-μL reaction mixtures. Both reaction mixtures and PCR conditions followed those in previous study [29]. The amplified products were purified and sequenced using ABI prism 3730 Genetic Aanlyzer (PE Applied Biosystems, Foster, CA, USA). The accession numbers are MH428836-MH428843.

Based on the results of Alvarado *et al*. [30], a total of 16 ITS sequences retrieved from GenBank and aligned with the eight ITS sequences amplified from this study by BioEdit 5.0.6 [31] and Clustal X [32]. Two species *Clitocybe favrei* (GU234009) and *C*. *vibecina* (GU234049) were used as the outgroup. The data matrice for ITS sequences analysis was produced. Bayesian analysis was conducted with MrBayes v.3.1.2 [33]. The best-fitting model of sequence evolution was chosen by MrModelTest v.2.2 [34]. The Bayesian analysis was run, under the GTR model, with four chains, and trees sampled every 500 generations. The average split frequencies were checked to determine optimal convergence of the chains below 0.01 after 2,000,000 generations. The first 25% of the sample trees was designated as burn-in, and the remaining samples were retained for further analyses. The topologies were used to generate a 50% majority-rule consensus tree for posterior probabilities (PP).

### ISSR and SRAP amplification

A total of 14 primers that produced clearly distinguishable and reproducible fragments were selected and used in this study for ISSR and SRAP analyses (Table 2). All of the amplification reactions were performed in a PCR Amplifier (BIO-RAD S1000, Hercules, CA, USA) in 25-μL reaction mixtures. For the ISSR analysis, the reaction mixture contained 12.5 μL of 2×Power TapPCR Master Mix (0.2 mM deoxynucleoside triphosphates, 4.0 mM MgCl_2_, and 2.5 U of Taq DNA polymerase), 1 μL of primer, 1 μL of template DNA, and 10.5 μL of ddH_2_O. The amplification included an initial denaturation at 94°C for 2 min; followed by 37 cycles of 35 s at 94°C, 45 s at 42–60°C, and 90 s at 72°C; and a final extension of 10 min at 72°C. For SRAP analysis, the reaction mixtures contained 12.5 μL of 2×Power TapPCR Master Mix, 1 μL of each primer, 1 μL of template DNA, and 9.5 μL of ddH_2_O. The amplification included an initial denaturation at 94°C for 5 min; followed by 35 cycles of 1 min at 94°C, 1 min at 50°C, and 1 min at 72°C; and a final extension of 10 min at 72°C. All of the PCR products were separated by electrophoresis on a 1.5% agarose gel with 1 × TBE buffer at 80 V for 3 h. The gels were stained with ethidium bromide and photographed under ultraviolet light (Bio-Rad ChemiDoc XRS, Hercules, CA, USA). The analyses were repeated at least twice, and molecular weights were estimated using a DNA marker (DNA Marker 2000, TIANGEN Biotech Co., Ltd., Beijing, China).

**Table 2:** ISSR and SRAP primer sequences for *Lepista nuda*.

Molecular marker	Primer	Sequence (5'→3')
ISSR	J1	(AC)_7_T
	J2	(CAC)_4_RC
	J11	(CA)_6_AC
	J14	(GGGTG)_3_
	J16	(GA)_8_C
	J17	(GA)_8_T
SRAP	Me2/Em4	TGAGTCCAAACCGGAGC/GACTGCGTACGAATTTGA
	Me2/Em7	TGAGTCCAAACCGGAGC/GACTGCGTACGAATTCAA
	Me10/Em1	TGAGTCCAAACCGGAAA/GACTGCGTACGAATTAAT
	Me10/Em11	TGAGTCCAAACCGGAAA/GACTGCGTACGAATTCTA
	Me10/Em13	TGAGTCCAAACCGGAAA/GACTGCGTACGAATTCTG

### Data analysis

Image Lab software (Bio-Rad ChemiDoc XRS, Hercules, CA, USA) combined with visual assessment was used to score DNA bands on the gels as “1” for present or “0” for absent, which generated a binary matrix. To increase the number of genetic loci, the DNA bands produced by ISSR and SRAP markers were combined. Genetic diversity analysis was performed using POPGENE version 1.31 [35]. Components of genetic variance within and among populations were estimated by analysis of molecular variance (AMOVA) using GenAlEx version 6.5 [36]. The correlation between population genetic distance and geographic distance was assessed by Mantel tests using the TFPGA version 1.3 [37]. Finally, a UPGMA dendrogram was constructed by using NTSYS-pc version 2.10 [38].

### Antioxidant activity analysis

One represented sample was randomly selected from each location and the dried mushrooms were ground into a fine powder by a crushed mill, and then were through a 0.15-mm sieve for further analysis. Mushroom powders were extracted by using distilled water for 3 hours at 75°C. After centrifuging, the extracting solution was tested the antioxidant activity. The scavenging activity of the water extracts from each sample on DPPH radicals was measured according to the method of Cheung *et al*. [39] with some modifications. We tested concentration (40 μg/mL) of mushroom water extract and used water to instead of mushroom water extract as a control. We used vitamin C as a standard. The reaction mixture was vortex mixed at room temperature and the absorbance (Abs) was measuring at 517 nm with a spectrophotometer. The scavenging activity of the DPPH radical was calculated using the following equation: Scavenging activity (%) = 100 × (A_Control_—A_Sample_)/A_Control_, where A _Control_ is the absorbance of the control reaction (containing all reagents except the test extract) and A _Sample_ is the absorbance of the test compound.

## Results

### Identification of samples

A phylogenetic tree based on ITS region showed that the eight sequences from this study and *L*. *nuda* formed a monophyletic clade with strong support (PP = 0.96). The result confirmed that the samples used in this study were the species of *L*. *nuda* (S1 Fig).

### Genetic diversity of *L*. *nuda* populations

As noted earlier, POPGENE software was used to analyze the combined ISSR and SRAP data. The results showed that the number of polymorphic bands in the eight populations ranged from 63 to 127 and that the percentage of polymorphic bands ranged from 29.4 to 59.4%, with an average of 47.6% (Table 3). Among all 72 samples, the number of polymorphic bands, the percentage of polymorphic bands, Nei’s genetic diversity index, and Shannon information index were 202,94.4%,0.3411,and 0.5042, respectively. Among the populations, genetic diversity was highest for the YC population (He = 0.2487, I = 0.3581) and lowest for the CBS population (He = 0.1291, I = 0.1835).

**Table 3:** Genetic diversity of *Lepista nuda* populations as indicated by ISSR and SRAP.

Population	A	PPB	He	I
MDJ	102	47.66	0.1691 (0.024)	0.2510 (0.034)
NI	76	35.51	0.1582 (0.026)	0.2250 (0.036)
YC	127	59.35	0.2487 (0.026)	0.3581 (0.036)
CBS	63	29.44	0.1291 (0.024)	0.1835 (0.034)
CC	102	47.66	0.1832 (0.024)	0.2684 (0.035)
BX	122	57.01	0.2298 (0.025)	0.3333 (0.046)
HR	115	53.74	0.2162 (0.025)	0.3149 (0.036)
SY	108	50.47	0.1979 (0.025)	0.2873 (0.036)
Population level	101.88	47.61	0.1915 (0.022)	0.2777 (0.032)
Species level	202	94.39	0.3411 (0.019)	0.5042 (0.024)

A: Number of polymorphic bands; PPB: the percentage of polymorphic bands; He: Nei’s genetic diversity index; I: Shannon’s information index.

The AMOVA revealed significant genetic differences among populations (Table 4), which was consistent with the Nei’s genetic diversity analysis. The highest percentage of the total genetic variance was the variance within the separate populations (42%). The genetic variance was 29% both among regions and among populations within regions. The gene flow (Nm = 0.6265) between the populations of *L*. *nuda* was estimated by the AMOVA analysis.

**Table 4:** Summary of the AMOVA results for 72 samples of *Lepista nuda*.

Source of variance	d.f.	SS	MS	Estimated variance	Percentage	Phi statistic	Value	P
**Among Regions**	2	776.953	388.477	12.715	29	**PhiRT**	0.292	0.001
**Among Pops**	5	652.298	130.460	12.591	29	**PhiPR**	0.408	0.001
**Within Pops**	64	1167.402	18.241	18.241	42	**PhiPT**	0.581	0.001
**Total**	71	2596.653		43.546	100			

d.f., degrees of freedom; SS, sum of squares; MS, mean sum of squares; PhiRT, PhiPR, and PhiPT indicate the proportion of the total genetic variance that is due to the variance between regions, the variance among populations within a region, and the variance among individuals within the species, respectively.

### Genetic distances between the populations

**Fig 2:**
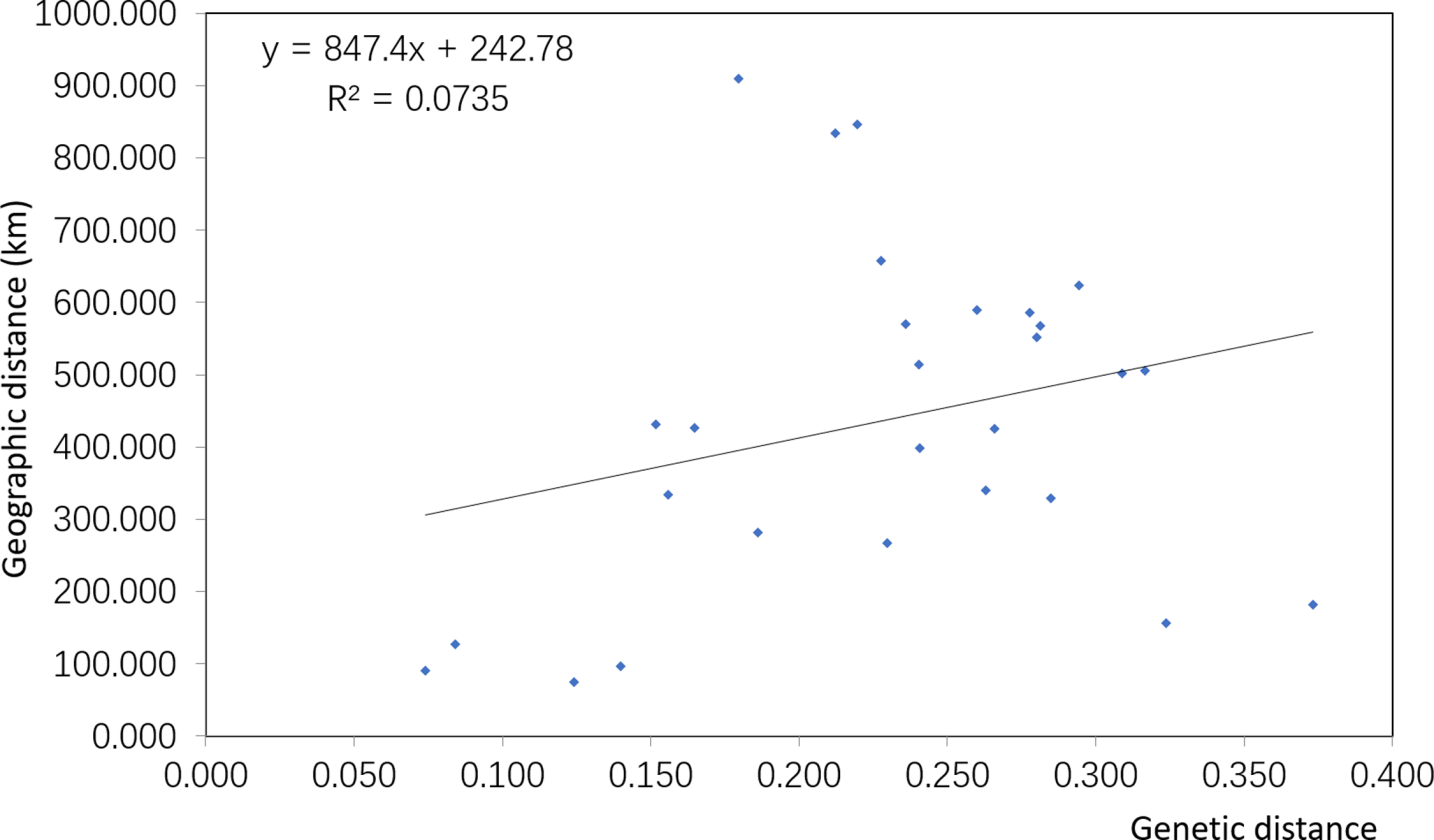
Relationship between geographic distance and genetic distance within the eight populations of *Lepista nuda*.

The genetic distances between the eight populations of *L*. *nuda* ranged from 0.0740to 0.3731 (Table 5). The genetic distance was smallest between the BX and the HR population, which are located in the same region, and was largest between the MDJ population and the CBS population, which are located in Heilongjiang Province and Jilin Province, respectively. The Mantel test indicated an absence of significant correlation between genetic distance and the geographical distance (r = 0.271; P = 0.12) (Fig 2).

**Table 5:** Nei’s genetic distance of *Lepista nuda* populations based on ISSR and SRAP.

Population	MDJ	NI	YC	CBC	CC	BX	HR	SY
MDJ	0							
NI	0.1242	0						
YC	0.1647	0.1517	0					
CBS	0.3731	0.3237	0.2777	0				
CC	0.2406	0.2849	0.2358	0.2630	0			
BX	0.2277	0.2599	0.1796	0.2403	0.1559	0		
HR	0.2814	0.3165	0.2196	0.2658	0.1861	0.0740	0	
SY	0.2943	0.2800	0.2122	0.3088	0.2297	0.1399	0.0840	0

### Cluster analysis

**Fig 3:**
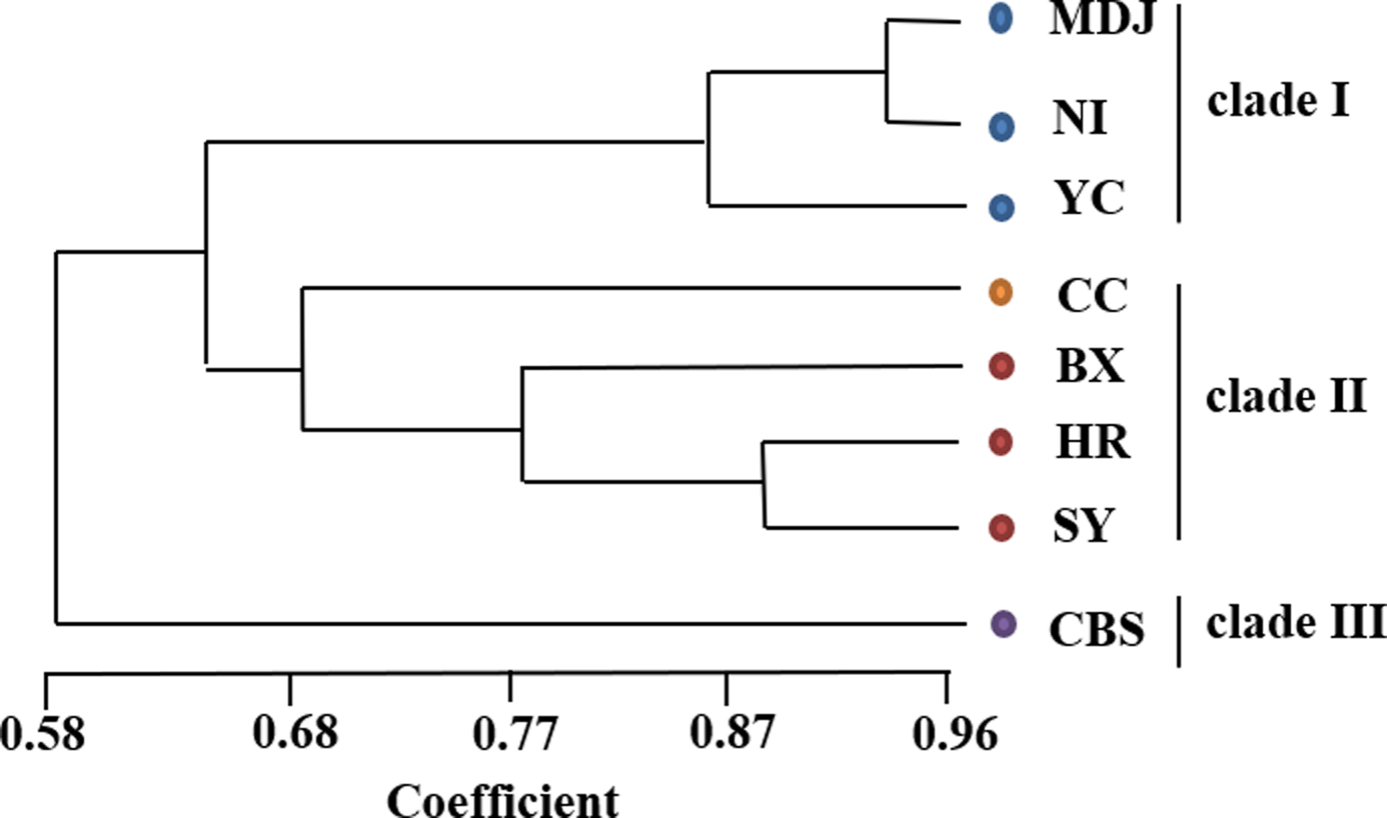
UPGMA dendrogram of the eight populations of *Lepista nuda*.

The ISSR and SRAP data were combined and analyzed by NTSYS software to generate UPGMA dendrogram (Figs 3 and S1). When the similarity coefficient was set at 0.64, the eight populations of *L*. *nuda* formed three clades. The three populations in clade I were from Heilongjiang Province, and the five populations in clade II were from Jilin and Liaoning Provinces. The one population in clade III was from Changbai Mountain.

### Antioxidant activity analysis

**Fig 4:**
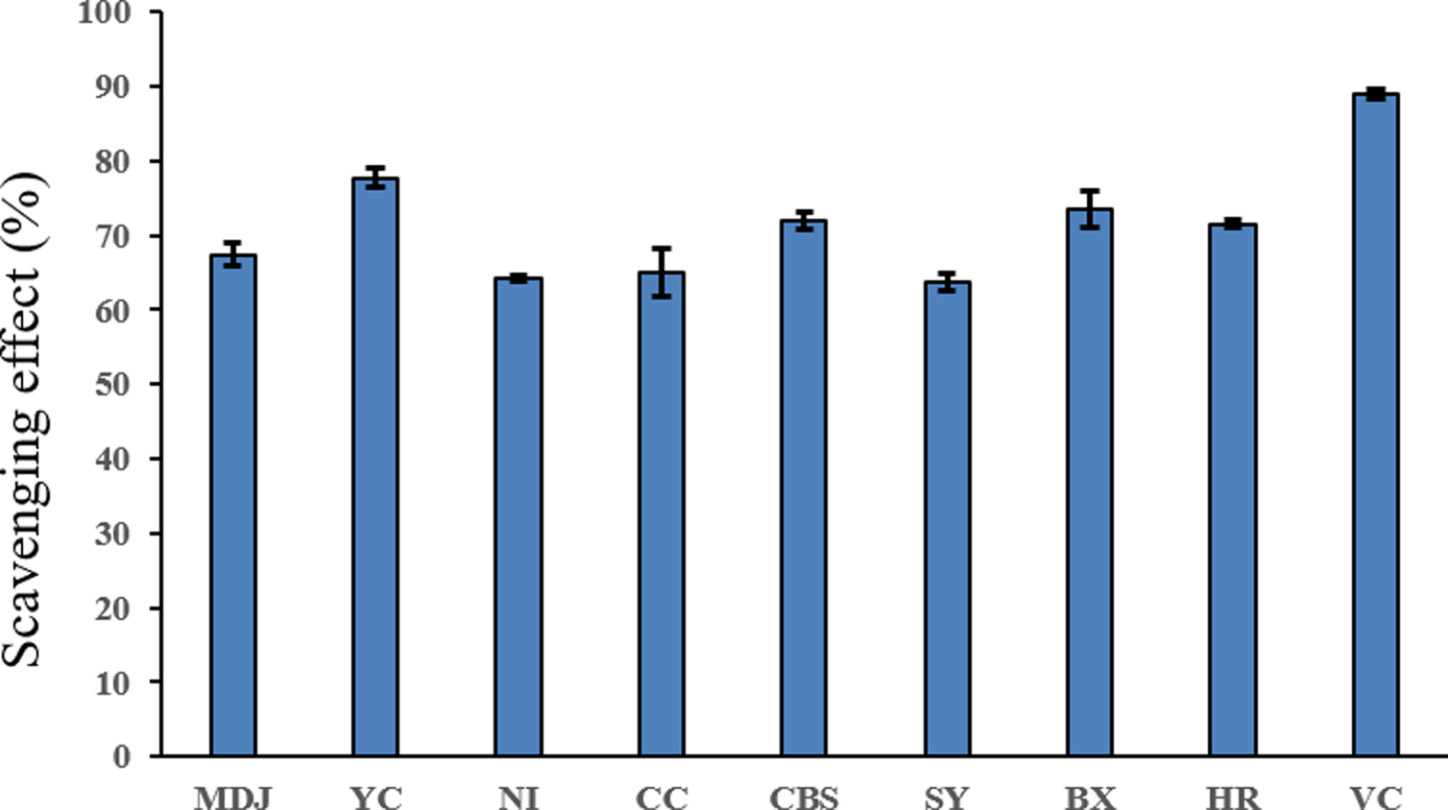
Column diagram analysis for antioxidant activity on water extract of each population. Vitamin C was as a standard.

The free radical scavenging of DPPH can be used to evaluate the antioxidant activity of extracts. In Fig 4, water extracts of eight samples exhibited more than 60% DPPH scavenging activities, indicating that all of the *L*. *nuda* samples had efficient antioxidant activity. The scavenging effects of water extracts from each population and standard on the DPPH radical decreased in the order of V_C_>YC>BX>CBS>HR>MDJ>CC>NI>SY and were 88.9, 77.7, 73.5, 72.0, 71.5, 67.4, 65.0, 64.2 and 63.8 at the concentration of 40 μg/mL, respectively.

## Discussion

SRAP molecular marker were originally developed for gene tagging in *Brassica oleracea* L. to specifically amplify coding regions of the genome [12]. SRAP have application in the fields of systematics, conservation, biogeography, and ecology, because they are easy to develop and use, are inexpensive, required small amounts of tissue, and can be used to detect high levels of polymorphism. For fungi, SRAP was initially used to analyze the genetic diversity of *Ganoderma* strains [40], then it was applied successfully in the studies of genetic diversity of some edible mushroom [13–15, 17, 18]. In this study, the genetic diversity of 72 samples of the wild edible mushroom *Lepista nuda* from Northeast China was investigated by use of ISSR and SRAP molecular markers. Six single primers for ISSR and five primer pairs for SRAP revealed a high level of genetic variation among the 72 samples. A total of 214 loci were found in the eight populations of *L*. *nuda*. Among them, 202 loci were polymorphic (91 loci for ISSR; 111 loci for SRAP), and the percentage of polymorphism was as high as94.4%. The results showed that the average polymorphic loci of each SRAP primer pair was much higher than some other species (22 loci for *L*. *nuda* vs. 12 loci for *Lentinula edodes* [14] and 8 for *Pleurotus citrinopileatus* [15]). The average polymorphic loci generated by each ISSR primer of *L*. *nuda* (15 loci) were similar to the other species, such as *L*. *edodes* (14 loci) [14] and *P*. *citrinopileatus* (8 loci) [15]. Therefore, the ISSR and SRAP markers were found to be useful tools for studying the genetic diversity of *L*. *nuda*.

Generally, gene flow was low when Nm value was less than 1.0 [41]. In this study, the Nm value was 0.6265, which shows that the gene flow of *L*. *nuda* in Northeast China is weak. In comparison, the gene flow of *Pleurotus eryngii* var. *tuoliensis* was found to be 1.794 [42]. The weak gene flow of *L*. *nuda* has at least two possible explanations. First, the species usually grows during the rainy season, and rain might limit the spread of spores. Second, habitat fragmentation in Northeast China is likely to reduce the exchange of spores between populations.

The genetic distance between eight *L*. *nuda* populations was not correlated with their geographical distance. For example, the geographic distance was greatest between the YC population and BX population (909 km), but the genetic distance was highest between the MDJ and the CBS populations (0.3731). The results also showed that all of the genetic distances between the CBS population and the other populations were very high. A similar phenomenon was reported for the plant *Liparis japonica* in Northeast China [43]. This lack of significant correlation between geographic and genetic distance might be explained by Changbai Mountain, which is believed to have been a refuge in the Last Glacial Maximum [44, 45]. A refuge in Changbai Mountain would enable the long-term survival of isolated *L*. *nuda* populations and might thereby promote the development of genetic differences unrelated to geographic distance.

Although *L*. *nuda* currently has a wide geographical distribution in Northeast China, the species may be endangered. On the one hand, the populations are small, and the habitat is increasingly fragmented. On the other hand, the results of this study indicate that gene flow among populations is low. We therefore suggest that measures are needed to protect the genetic resources of the species. We suggest that harvesting of *L*. *nuda* in Northeast China should be limited. It might also be useful to collect and store basidiomata as a gene bank for the species. The introduction of basidiomata from other places could increase the number of genotypes. Finally, domestication of *L*. *nuda* could reduce the harvesting of wild populations.

In the present study, the samples of each population showed antioxidant activity to some extent, while the trend of antioxidant activity diversity is not consistent with either the genetic diversity or the geographic distribution of each population. Samir and Mathilde [46] obtained a similar result in the study of Lingonberry. They suggested that the possible reason for the disagreement between the chemical and molecular diversity was that the noncoding genes of genome are not accessible to the expression of antioxidant activity. Fungi are able to produce many secondary metabolites with antioxidative activities including a number of phenolic compounds, ascorbic acid and so on [47]. The production of these antioxidant agents usually affected by the environmental factors, such as pH value and nutritional conditions [48]. Therefore, the differences in the collection time and location of the samples might have resulted in the inconsistent between the molecular and chemical diversity.

## Supporting information

S1 Fig Fifty percent majority-rule Bayesian cladogram based on ITS sequence analyses. The node support is indicated by Bayesian posterior probabilities on branch. Only support values greater than 0.60 in Bayesian are shown.
(TIF)

S2 Fig UPGMA dendrogram of 72 samples of *Lepista nuda*. (TIF)
