# Supplementary figures and images for: Genetic diversity of Lepista nuda (Agaricales, Basidiomycota) in Northeast China as indicated by SRAP and ISSR markers
Source: PLoS One. 2018 Aug 27;13(8):e0202761. doi: 10.1371/journal.pone.0202761 (PMC6110484; doi:10.1371/journal.pone.0202761)

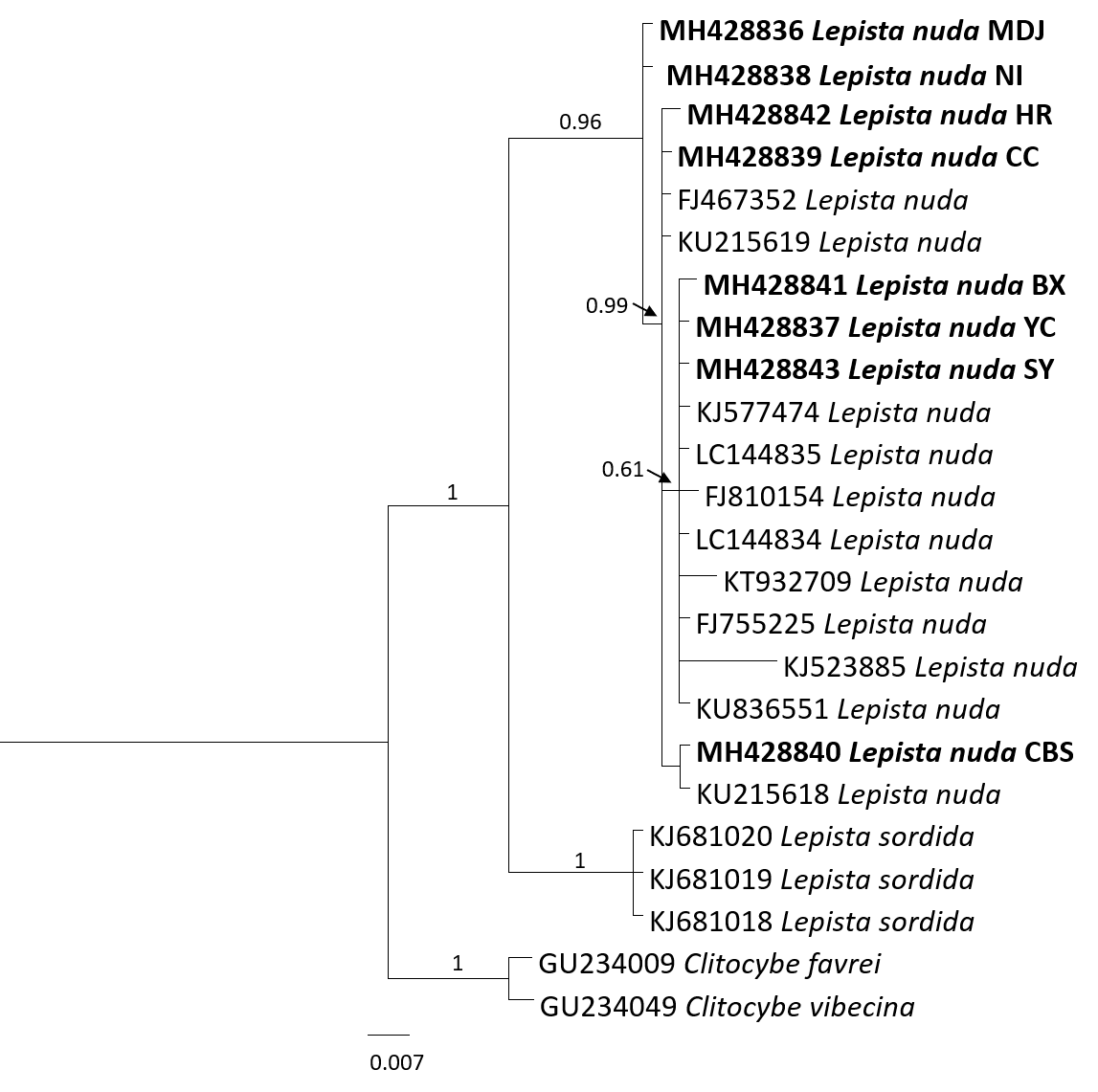

Supplement: S1 Fig — The node support is indicated by Bayesian posterior probabilities on branch. Only support values greater than 0.60 in Bayesian are shown. (TIF) [file pone.0202761.s001.tif]

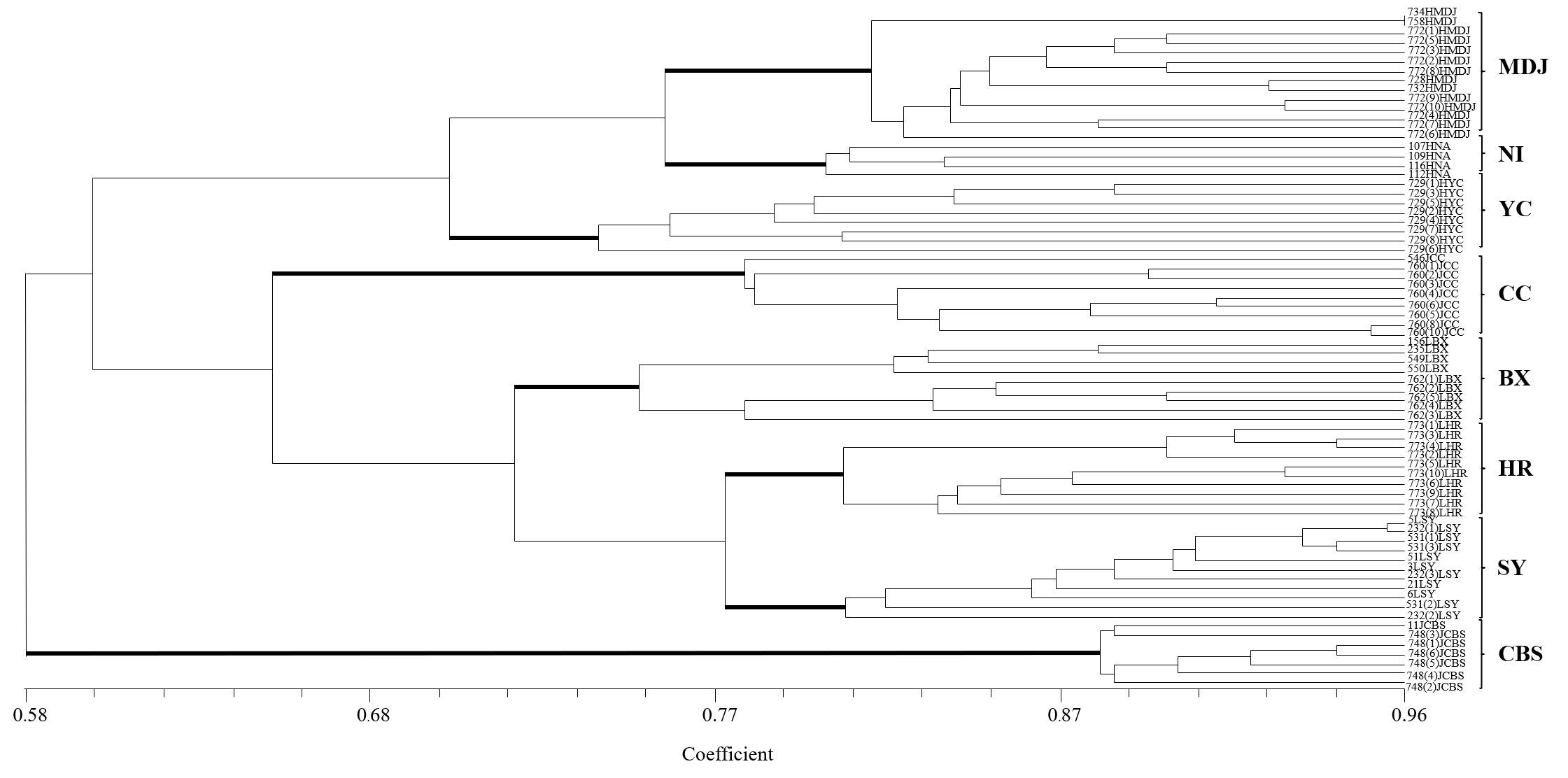

Supplement: S2 Fig — (TIF) [file pone.0202761.s002.tif]
